# Targeted Therapy in Rheumatoid-Arthritis-Related Interstitial Lung Disease

**DOI:** 10.3390/jcm12206657

**Published:** 2023-10-20

**Authors:** Robert Harrington, Patricia Harkins, Richard Conway

**Affiliations:** St. James’s Hospital, LS9 7TF Dublin, Ireland; harkinp@tcd.ie

**Keywords:** RA, ILD, rheumatoid arthritis, interstitial lung disease, treatments, biologics, small molecules, disease modifying anti-rheumatic drugs, abatacept, rituximab

## Abstract

Rheumatoid arthritis (RA) is a chronic autoimmune multisystem inflammatory disease in which lung involvement is the most common extra-articular manifestation. Parenchymal lung involvement or interstitial lung disease (ILD) is a significant cause of morbidity and mortality and there is a paucity of evidence-based guidance on how to best treat RA-ILD. This review article aims to evaluate the evidence from cohort studies and best real word data from registries. Extensive discussion of the relative merits and drawbacks of glucocorticoids, various biologics, small molecules and anti-fibrotics is presented. The limited available guidelines in RA-ILD are also discussed and a rational treatment algorithm is offered.

## 1. Introduction

### 1.1. Prevalence, Etiology and Pathogenesis

Rheumatoid arthritis (RA) is a chronic autoimmune systemic inflammatory disease affecting 0.5–1.0% of the global population [1]. While synovial inflammation, joint pain and deformity are the hallmarks of RA, it is a true multi-system disease, with pulmonary disease now recognised as the second most common cause of death after cardiovascular disease [2]. It is believed that interstitial lung disease (ILD) in RA patients confers a three-fold increase in mortality risk compared with RA patients without lung involvement. Historically, the median survival was 3 years upon diagnosis [3]. Furthermore, when compared to the general population, RA patients are nine times more likely to develop ILD [4]. Early ILD may go unrecognised and clinically significant RA-ILD occurs in 3–8% of cases [3,5].

The recognised risk factors for RA-ILD development and poorer prognosis include age greater than 65, smoking, male gender, anti-citrullinated protein antibodies (ACPA), human leukocyte antigen HLA-DR4, a usual interstitial pneumonia pattern (UIP) of disease and subcutaneous nodules [6]. One postulated pathogenic explanation for RA-ILD development is that of the two-hit hypothesis, whereby disease may develop in those with genetic risk factors (e.g., certain HLA haplotypes, male gender) and exposed to an environmental trigger (e.g., cigarette smoke, silicone, other noxious occupational inhalants). This may cause the pathologic citrullination of peptides and proteins at the level of the alveolus, triggering T and B lymphocytes, which in turn lead to ACPA and immune complex production [7].

### 1.2. Patterns of RA-ILD and Treatment Response

Importantly, the histologic and high-resolution CT (HRCT) pattern of lung involvement has a significant impact on the prognosis and response to immunosuppressive treatment. The UIP pattern is seen in approximately half the cases of RA-ILD. The non-specific interstitial pneumonia (NSIP) pattern of disease is present in about a quarter of cases, followed then by the organising pneumonia (OP) pattern in about 10–15% of cases. The lymphoid interstitial pneumonia (LIP) pattern is seen rarely and tends to be treatment-resistant given that damage cannot be reversed on account of the cystic hollowed-out nature of parenchymal involvement. The natural history of the UIP pattern is similar to idiopathic pulmonary fibrosis and tends to be unrelenting if untreated. It is characterised by a HRCT appearance that is bilateral and predominantly basilar, with also subpleural fibrosis, cyst formation (honeycombing) and traction bronchiectasis. The NSIP pattern tends to be bilateral with peripheral ground-glass opacities, septal thickening and consolidation. Traction bronchiectasis may also be a feature; subpleural sparing is typical. The OP pattern features patchy but dense areas of lobar airspace consolidation on HRCT. There may be some ground-glass change surrounding these foci of consolidation. Lastly, the LIP pattern features thin-walled cysts with or without ground glass change [8,9,10,11].

**Figure 1:**
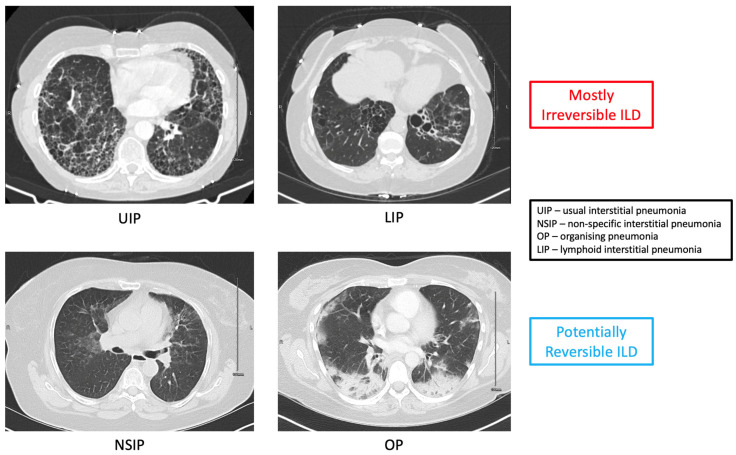
Patterns of ILD in RA.

As a general rule of thumb, the UIP and LIP patterns are considered irreversible, whereas the NSIP and OP patterns can resolve with glucocorticoids (GCs) and other immunosuppressive treatments. While damage cannot generally be reversed in UIP and LIP patterns, that does not mean there is no role for immunosuppressive treatment. On the contrary, the UIP pattern shows the stabilisation of both HRCT appearance and pulmonary function tests (PFTs) over time with certain treatments. This is a key distinction between the UIP pattern of disease seen in RA-ILD and the IPF-typical HRCT pattern of disease. While they have the same radiographic appearance, disease is responsive to both immunosuppression and anti-fibrotics in UIP RA-ILD, whereas anti-fibrotics are only able to slow deterioration in IPF. Both the NSIP and OP patterns of disease in RA-ILD respond very well to immunosuppression, showing the reversal or resolution of HRCT findings. The reason for this is that the bulk of the HRCT picture is likely inflammation in NSIP and OP, which can be reversed, whereas the typical findings on HRCT in UIP and LIP are reflective of fibrotic irreversible established changes. Taken from patients attending our centre, Figure 1 gives an illustrative overview of the main four patterns of disease and these concepts.

Despite the associated morbidity and mortality, there has been little consensus regarding guidelines for the treatment of RA-ILD patients. Unfortunately, there is little informed evidence by way of randomised control trials (RCTs) for potential treatments in RA-ILD. In terms of the hierarchy of evidence, most of the accepted evidence base comprises cohort, case–control, and cross-sectional studies. This review article aims to give a comprehensive review of these key studies so that practicing physicians are better informed of the possible treatment options.

## 2. Search Strategy

A systematic literature search for relevant articles was performed using PubMed, Embase and the Cochrane central register of controlled trials. The search was performed with no date limits and was last updated on 26 September 2023. The keywords ‘rheumatoid arthritis’ AND ‘interstitial lung disease’ OR ‘RA-ILD’ AND (‘treatment’ OR ‘therapeutics’) were used. The reference lists of key articles were also reviewed.

## 3. Immunosuppressive Therapeutics

While one class of RA therapeutic may have a comparable effect to another on synovial disease, the same is not necessarily true when it comes to pulmonary disease. An in-depth exploration of each drug class and their beneficial, or perhaps deleterious, effect on ILD stabilisation is provided. The evidence base dispelling the notion that methotrexate (MTX) exacerbates RA-ILD is highlighted and our preferred treatment strategy, which combines MTX with more modern agents, is offered. The relative merits of each therapeutic immunosuppressive agent are detailed in Table 1.

### 3.1. Glucocorticoids

There remains much confusion regarding the role of GCs in the treatment of RA-ILD. The British Thoracic Society (BTS) produced the first guidelines for RA-ILD back in 2008. This recommended the use of prednisolone at 0.5 mg/kg/day for 1–3 months as the first-line treatment. It recommended a taper to less than 10 mg/day, with the addition thereafter of a disease-modifying anti-rheumatic drug (DMARD) [12]. Of course, the sequalae of long-term steroids cannot be overstated and the risk of infection with high doses is very real. In particular, the risk of pneumocystis jiroveci pneumonia (PJP) is particularly worrying, as this can cloud the clinical picture in RA-ILD patients.

One case–control study evaluating the effect of oral GCs on RA-ILD mortality found that patients treated with prednisolone for more than 3 months had a greater mortality risk than those who did not receive prednisolone, with a relative risk (RR) of 2.06. The RR from respiratory causes was greater still at 2.75. The authors do note however that the preponderance of the usual interstitial pneumonia (UIP) pattern in the treatment group may explain the greater mortality seen [13]. Similarly, the PANTHER trial, which evaluated the effect of combination prednisolone, azathioprine (AZA) and N-acetylcysteine on forced vital capacity (FVC) in idiopathic pulmonary fibrosis (IPF), was terminated early due to increased morbidity and mortality in the AZA and prednisolone treatment arm [14]. Much of the excess mortality was due to respiratory tract infections. This is an important finding regarding glucocorticoids in frail UIP pattern ILD patients, and likely translates to the RA-ILD population as well.

The findings from Scott et al.’s study and the PANTHER trial contrast with the results of a retrospective case–control study performed by Song et al.; this study found that treatment with steroids, either as monotherapy or in combination with other immunosuppressants in 84 RA-ILD patients with a UIP pattern, improved or stabilised disease in 50% of the cohort. However, there was no statistically significant survival benefit compared with the untreated group despite the numerical trend [15]. Nevertheless, the consensus opinion is that glucocorticoids are initially useful in arresting the progression of ILD for NSIP, OP, and potentially lymphoid interstitial pneumonia (LIP), buying time for the long-lasting effects of other immunosuppressants to work.

The use of short-term steroids in combination with MTX and biological DMARDs (bDMARDs) may be reasonable on a case-by-case basis in NSIP, OP or LIP patterns of disease where the trajectory of progression is rapid and the patient cannot afford to wait for long-lasting immunosuppressants to take effect.

### 3.2. Methotrexate

MTX remains the anchor DMARD and the cornerstone of treatment in RA. Concern over a potential link between MTX and pulmonary toxicity first arose in 1983 and took many years to clarify [16]. The key studies that delve into the true nature of MTX and its role in RA-ILD are the ERAN and ERAS studies, which recruited over 2700 RA patients from England, Wales and Ireland. This large multicentre prospective cohort trial, with a follow-up period of up to 25 years in some patients, compared the prevalence of RA-ILD in MTX-exposed and non-MTX-exposed groups. The authors demonstrated that in the MTX exposed group, 97.5% (n = 1539) remained ILD-free, whereas in the non-MTX exposed group, 95.2% (n = 1061) remained ILD-free [5]. Not only does this dispel the idea that MTX is causative in RA-ILD development, but it also serves to illustrate the protective effect of MTX against RA-ILD development.

Further supportive evidence regarding the inverse relationship between MTX exposure and RA-ILD comes from Juge et al., as data from their derivation and validation samples showed a combined adjusted odds ratio (OR) of 0.43 (95% CI 0.26–0.69; *p* = 0.0006). In patients with RA-ILD, ILD detection was significantly delayed in the MTX ever-users compared to never-users (11.4 +/− 10.4 years and 4.0 +/− 7.4 years, respectively; *p* < 0.001) [17]. The findings of a smaller retrospective Mexican study from Rojas-Serrano et al. reinforce this idea. Adjusted for confounding variables, the survival of RA-ILD patients was higher with MTX (median 70 months) than without MTX [18]. Lastly, a meta-analysis of 22 studies with over 8500 patients showed that while MTX treatment results in a small increased risk of total respiratory complications due to the increased risk of infections, it does not increase the risk of non-infectious respiratory complications or death from lung disease. While largely reassuring, the authors do note however that a subgroup analysis of studies in which pneumonitis was described revealed an increased risk associated with methotrexate (RR 7.81, 95% CI 1.76–34.72) [19]. The CIRT Trial sheds further light on the potential risk of acute pulmonary toxicity with MTX. It originally intended to evaluate the potential protective role of MTX against atherosclerosis and cardiovascular disease in a high-risk cohort. CIRT randomly assigned 4786 patients to a low-dose MTX (median dose 16 mg weekly) or placebo group. The median follow-up was 23 months. The authors report seven (0.3%) cases of possible pneumonitis in the low-dose MTX group and only one (0.04%) in the placebo group (HR 6.94, 95% CI 0.85–56.0; *p*-value 0.04 by exact test). They also report that no cases could be considered probable or definite as too little information was provided [20]. While protective against RA-ILD, MTX can very rarely cause a drug-induced pneumonitis, although the absolute risk is very low at approximately 0.3%.

There is a large body of evidence to support the idea that MTX is protective against RA-ILD development and progression. As general guidance, the use of MTX as a first-line treatment in RA-ILD is a reasonable evidence-based approach. However, those with a poor baseline pulmonary reserve, who are unlikely to tolerate MTX pneumonitis, should not be started on the drug for arthritis in the first instance.

### 3.3. Tumour Necrosis Factor Inhibitors

The controversy over the role of tumour necrosis factor inhibitors (TNFi) in RA has continued for the past 20 years. The pioneering RCTs of TNFi in RA were powered and designed to demonstrate efficacy in reducing the articular and synovial components of disease, not the stabilisation of RA-ILD or the effect on incident ILD. Consequently, the highest yield data comes from large biologic registries. Data from the British Society for Rheumatology Biologics Register (BSRBB) were analysed by Dixon et al. They report an overall prevalence of ILD of 2.6% in RA patients from the register. The prevalence of ILD was significantly higher in the TNFi-treated patients at 2.9% (299/9294) compared with 1.8% (68/2454) in the conventional DMARDs (csDMARD)-treated patients. Despite this, there was no significant difference between groups in terms of the standardised mortality or ILD mortality, although ILD mortality was numerically higher with TNFi treatment (15/70) than with csDMARD treatment (1/14) (21% vs. 7%) [21]. Given the strict biologic initiation guidelines in the United Kingdom, a possible confounder here is that those that received TNFi treatment likely had a history prior to this of more active RA disease; this may account for some of the increase in ILD prevalence seen with TNFi treatment relative to csDMARD. In other words, perhaps historically these were more active patients with a higher inflammatory burden and possibly more resultant lung involvement. Nevertheless, the BSRBB findings are a cause for caution when selecting the most appropriate bDMARD in patients with known RA-ILD.

The BSRBB also provided some very insightful information on the relative efficacy of TNFi versus rituximab (RTX) in RA-ILD patients. Druce et al. compared the 5-year mortality rates in 43 RA-ILD patients treated with RTX as a first-line treatment with 309 RA-ILD patients treated with TNFi as a first-line treatment. Adjusting for confounders, RTX compared more favourably to TNFi, with a 5-year mortality hazard ratio (HR) of 0.53 [22]. While still not statistically significant, this cohort study suggested a trend towards a longer survival with RTX as a first-line treatment as opposed to the TNFi class.

The Japanese experience is similar. Nakashita et al. performed a prospective case–control study of 58 RA-ILD patients. The incidence of ILD exacerbation with TNFi versus non-TNFi biologics was statistically significantly higher, with 30.4% (14/46) in the former and 0% (0/12) in the latter (*p* = 0.024). Notably, of the 12 non-TNFi biologic patients, 9 were treated with tocilizumab (TCZ) and 3 with abatacept (ABA) [23]. This strongly suggests that alternative bDMARDs should be considered in RA-ILD patients. Nakashita’s group also performed a single-centre case–control study of 163 RA patients starting bDMARDs. Of the 163 patients, 58 were found to have ILD at baseline on CT. An ILD event was defined as either the emergence of ILD or the progression of ILD. Significantly, more ILD events occurred in the TNFi group than the non-TNFi bDMARD group; 88% vs. 60% *p* < 0.05. Amongst the non-TNFi group, TZC- and ABA-treated patients did not differ in this regard. Of the 58 RA patients with pre-existing ILD, 14 progressed, all of which were in the TNFi group [24].

Whether or not TNFi treatment increases the risk of RA-ILD development remains not entirely clear. What is almost certain at this point is that non-TNFi bDMARDs such as ABA, RTX and perhaps TCZ are better treatments to use when attempting to arrest RA-ILD progression. As a result, it is the author’s opinion that the treatment should be optimised by switching from TNFi to ABA or RTX in any RA patient with evidence of clinically significant ILD or progression on TNFi.

### 3.4. Rituximab

The bulk of the early evidence base for B-cell depletion and the use of RTX in RA-ILD comes from three cohort studies: two prospective and one retrospective. The first comparative cohort study in which RTX was compared with the TNFi class comes from the UK BSRBB. This prospective observational comparative study, conducted between 2008 and 2017, screened 1632 RTX and 15,644 TNFi patients. It compared mortality in patients with RA-ILD who received RTX or TNFi as their first biologic. Only 45 RTX patients and 309 TNFi patients met the RA-ILD inclusion criteria. As discussed earlier, the adjusted 5-year mortality in rituximab-treated patients is approximately half that of the TNFi-treated patients (HR 0.53, 95% CI 0.26 to 1.10), but the difference was not statistically significant [21]. Nevertheless, this study provided some insight into the potential beneficial role of rituximab in RA-ILD treatment.

The second landmark UK paper investigating the role of rituximab in RA-ILD treatment was also published in 2017. This large single-centre retrospective observational cohort study screened 700 RA patients treated with RTX. In total, 56 patients with ILD were identified and 44 had available PFT data. The progression of ILD was defined as a decrease in pre-RTX (FVC) > 10% or a predicted decrease in the diffusion capacity of carbon monoxide (DLCO) > 15%. In total, 23/44 (52%) remained stable and 7/44 (16%) improved. In total, 14/44 (32%) progressed despite RTX treatment. In total, 9/56 (16%) of the cohort died during follow-up from progressive ILD. Unsurprisingly, perhaps, the progressors tended to have a UIP pattern and more severe ILD pre-treatment, with a predicted median DLCO of 42%. This cohort study demonstrated long-term efficacy with RTX treatment in terms of stabilising RA-ILD for many [25].

A longitudinal multi-centre study from the Spanish NEREA registry published in 2020 also added to the RTX evidence base. This defined a very tight pulmonary “functional impairment” (FI) as a decline in FVC of ≥5%. Of the 68 RA-ILD patients, 31 received RTX. In total, 50% reached FI within 1.75 years of ILD diagnosis. However, multivariate analysis showed that RTX reduced the risk of FI by half compared with non-exposure (HR 0.51, 95% CI 0.31–0.85). In terms of the RTX non-exposed group, the authors note that this group were treated with azathioprine (AZA), ABA and mycophenolic acid (MMF) in descending order of frequency. The take home message here is that RTX-exposed patients have a much higher probability of stabilising or improving compared with other forms of treatment [26].

A recent systematic review and meta-analysis examining the evidence for RTX use in RA-ILD identified 15 suitable studies in total: 11 retrospective and 4 prospective studies. Pooling the patients provided a total of 314 RTX-treated RA-ILD cases, with a heterogenous spectrum of disease representative of real-world clinical practice. The overall pooled proportion of patients with stabilisation or improvement in ILD was 0.88 (95% CI 0.76–0.96, *p* = 0.02). RTX improved the FVC from baseline by 7.5% (95% CI 1.35–13.65; *p* = 0.02, fixed effect). The authors report that while their meta-analysis highlights a significant improvement in the FVC after one year of RTX treatment, the trend towards an improvement in DLCO is not statistically significant. They conclude that there remains an urgent need for further studies, including RCTs comparing the effectiveness of RTX against other immunosuppressants [27].

### 3.5. Abatacept

Early real-world experience with ABA showed promising results in RA-ILD patients. ABA has been available in Japan since 2010 and Nakashita et al. performed a retrospective single-centre cohort study comprising 16 ABA-treated RA-ILD patients and 46 TNFi-treated RA-ILD patients. They found that none of the ABA-treated patients showed HRCT evidence of progression one year after starting ABA treatment. This is in contrast to the TNFi group, in which 30.4% (14/46) of patients showed evidence of ILD progression on HRCT (*p* < 0.013) [28]. While this is undoubtedly a short follow-up period to draw meaningful conclusions from, this study did suggest at the time that ABA was a promising therapeutic in RA-ILD. Furthermore, robust supportive evidence of this beneficial effect of ABA emerged from Japan in 2019. Mochizuki et al. published a retrospective cohort study comprising 131 RA patients treated with abatacept. In total, 58% had no ILD at baseline on HRCT, and this was referred to as grade 0. In total, 24% had grade 1 involvement, or the fibrosis of less than 1/3rd of the total lung volume on CT. In total, 14% had grade 2, or the fibrosis of between 1/3rd and 2/3rds of the total lung volume and 4% had grade 3, i.e., more than 2/3rds of the lung volume fibrosed. They defined deterioration as progression from one grade to the next. They found that there was only a progression rate of 8.4% with abatacept treatment over an almost 4-year period [29].

The Italian experience with ABA mirrors the early Japanese cohort studies. Cassone et al. published their retrospective multi-centre cohort study involving 44 RA-ILD patients treated with ABA in 2020. Serial PFTs found that the FVC and DLCO remained stable or improved in 86% and 91% of patients over a median follow-up time of 26.5 months. The HRCT appearance also remained stable or improved in 81% of patients. Additionally, they noted that the previous or concurrent use of MTX had no effect on the outcome [30].

Further evidence from Spain also shows a strong stabilising effect with ABA. Fernandez-Diaz et al. published a large national multi-centre observational study involving 263 RA-ILD patients treated with ABA. At baseline, this cohort had a median duration of ILD of 1 year, a predicted mean FVC of 85.9% and a predicted DLCO of 65.7%. After a median follow-up period of 1 year, the FVC and DLCO were stable in 87.7% and 90.6% of the cohort. The HRCT findings were stable in 76.6% of the cohort [31]. Further analysis from the same cohort showed that ABA is equally effective in stabilizing dyspnoea, lung function and radiological impairment in both UIP and NSIP patterns of RA-ILD alike [32].

Cumulatively, the body of evidence for ABA’s role in RA-ILD treatment is amongst the strongest compared with other classes of immunosuppressant and should probably be considered alongside RTX as the first-line treatment.

### 3.6. Tocilizumab

TCZ is an interleukin-6 (IL-6) inhibitor that was first approved for RA in 2009 in Europe by the European Medicines Agency (EMA) and in 2010 in the United States by the Food and Drug Administration (FDA). The data supporting the use of TCZ in RA-ILD are somewhat mixed. Curtis et al. performed a large retrospective cohort study in RA to investigate the incidence of ILD in patients receiving TNFi, IL-6 inhibitors or other bDMARDs acting on T-cells or B-cells. They found no significant difference in the incidence of ILD or hospital admissions for patients with known RA-ILD in 59 TCZ-treated patients compared with 232 TNFi-treated patients [33]. Interestingly, Nakashita et al. found no increase in either incident ILD or the exacerbation of known ILD in 36 TCZ-treated RA patients. However, the same was not true of TNFi-treated patients. Of the 58 patients with pre-existing RA-ILD, 14 exhibited the progression of lung disease. Notably, all 14 progressors were receiving TNFi [23].

If Nakashita’s study was interpreted as a win for TCZ in comparison to the TNFi class, then the picture was clouded by a post-marketing surveillance study from Japan of 7901 TCZ-treated RA patients, as the incidence of ILD was found to be 10 cases per 1000 patients per year. This is approximately twice as common as their estimated incidence of the disease [34]. Is it the case that they underestimated the incidence of disease arising without treatment? Perhaps this may explain why TCZ appears in a less favourable light in this post-marketing surveillance study. To better investigate the role of TCZ further, the same authors, Otsuji et al., evaluated the efficacy of TCZ in 34 patients with known RA-ILD. They found that not only was RA disease activity significantly improved, but the Krebs von den Lungen-6 (KL-6) level, which is associated with ongoing lung inflammation and active fibrosis, was also significantly reduced at 2 and 6 months after TCZ administration. In the 22 patients who had a follow-up thorax CT at one year, none were deemed to have progressed [35].

While TCZ may confer a protective effect against ILD in RA patients, there is insufficient evidence to use it ahead of either ABA or RTX. Prospective comparative studies are still needed to determine its relative efficacy to both ABA and RTX.

### 3.7. JAK Inhibitors

Janus Kinase Inhibitors (JAKi) are oral novel small molecules and the newest class of RA therapeutics. To date, the paradigm for RA-ILD treatment has been tight disease control with the up-titration of MTX and the addition of bDMARDs such as ABA or RTX. More recently, small real-world cohort studies have suggested that the magnitude of the proposed beneficial effect of JAKi on RA-ILD is comparable to ABA in terms of HRCT improvement, stabilisation, and progression [36]. Additionally, the results of a UK retrospective study involving 28 JAKi-treated patients and 19 RTX-treated patients, all with RA-ILD and/or bronchiectasis, showed no difference between groups in terms of the risk of respiratory infections, ILD exacerbations and death from respiratory causes [37].

JAKi has also demonstrated a greater reduction in KL-6 levels than other bDMARDs. Given that elevated KL-6 levels tend to correlate with the active fibrotic process, it is hoped therefore that this will result in a strong stabilising effect on ILD with JAKi treatment [38].

The best supportive evidence for the potential beneficial role of JAKi in RA-ILD comes from a large retrospective cohort study involving over 28,000 RA patients in the United States. Patients treated with tofacitinib (TOFA) had the lowest incidence of ILD of all bDMARDs. After adjusting for covariates, TOFA still demonstrated a 69% reduced risk of ILD (adjusted hazard ratio 0.31; 95% CI, 0.12–0.78; *p* = 0.0090) compared with patients treated with ADA [39]. This is a notable reduction in incident ILD, and this result drives the need for a prospective RCT to better determine whether this effect is real and profound.

### 3.8. Mycophenolic Acid

Mycophenolic acid (MMF) in an anti-metabolite that inhibits T and B lymphocyte proliferation. It has been used since the early 1990s as a potent immunosuppressant in transplant medicine. It has been widely used in the treatment of connective-tissue-disease-related interstitial lung disease (CTD-ILD) for years, and particularly in diffuse systemic sclerosis lung (dSSc) involvement. The landmark Scleroderma Lung Study II (SLSII) showed that the treatment of SSc-ILD with MMF for 2 years or cyclophosphamide (CYC) for 1 year both resulted in significant improvements in pre-specified measures of lung function, dyspnoea, lung imaging, and skin disease over the 2-year course of the study. While MMF was better tolerated and associated with less toxicity, it was not more efficacious than CYC at the 2-year mark [40].

While it is tempting to assume that MMF will have a beneficial effect on RA-ILD given its efficacy in other rheumatic conditions, it may not be ideal to promote the drug as a first-line therapy in RA-ILD given that there are various bDMARDs with a more robust evidence base in this domain. To the best of our knowledge, there are only four publications in the literature and a small number of MMF-treated RA-ILD patients that suggest its beneficial role.

Saketkoo et al. described a small case series of just three patients with RA-ILD treated with MMF. The improvement in PFTs and HRCT stabilisation suggested a possible beneficial role [41]. More insightful is the 2013 publication of Fischer et al., which retrospectively analysed a cohort of 18 RA-ILD patients treated with MMF, with a median follow-up time of 2.5 years. They found that MMF was well tolerated and enabled the GC burden to be reduced. In terms of a meaningful effect on lung function, MMF seemed to arrest the deterioration in FVC. The trend towards improvement in this parameter did not reach statistical significance, however [42].

A retrospective UK study from 2016 reports a better survival rate for RA-ILD treated with MMF rather than with azathioprine (AZA). Notably, they found that the relative risk of death was increased with glucocorticoid (GC) treatment, unchanged in those treated with AZA, and reduced in those given MMF [43]. Lastly, Oldham et al. compared the use of AZA and MMF in patients in a sub-group of just 15 RA-ILD patients. The trend was towards the stabilisation of pulmonary function over time. The authors also highlight that there were more side effects in the AZA group [44]. Neither study is particularly convincing regarding the beneficial effect of MMF on RA-ILD, especially given that the PANTHER trial was terminated early on account of increased morbidity and mortality in the AZA and prednisolone groups [14]. We can only postulate that perhaps MMF is not as harmful as AZA or GCs in RA-ILD.

The take-home message that can be derived from the limited evidence base is that perhaps MMF has some stabilising effect on RA-ILD, but it is difficult to quantify the magnitude of the effect or compare it to other forms of immunosuppression used in RA on the basis of these findings. While we have various bDMARDs with a more robust evidence base, such as ABA and RTX, we should refrain from opting for MMF as a first-line treatment in RA-ILD. Prospective trials comparing ABA, RTX and MMF are needed to clarify this issue.

## 4. Anti-Fibrotics

The role of anti-fibrotics in the treatment of RA-ILD still requires further clarity. The current evidence base suggests that anti-fibrotics have a supportive synergistic role as an adjunct to safely maximising MTX and a suitable bDMARD such as ABA, RTX or a JAKi. The utility of nintedanib has already been demonstrated in idiopathic pulmonary fibrosis (IPF) in two replicate 52-week RCTs, INPULSIS-1 and INPULSIS-2 [45]. The INBUILD trial recruited 663 patients with progressive ILD to assess the efficacy of the drug in other forms of pulmonary fibrosis. In total, 170 out 663 patients were deemed to have autoimmune-disease-related ILD. Of particular interest to this review, a subgroup of 89 patients whose RA-ILD was treated with nintedanib or a placebo demonstrated a reduction in the rate of decline in forced vital capacity (FVC) over 52 weeks of 60%: −79.0 mL/year in the nintedanib group versus −196.9 mL/year in the placebo group. This equates to a difference of 117.9 mL/year with nintedanib versus the placebo (*p* = 0.041). The efficacy of nintedanib versus the placebo was numerically greater in RA-ILD patients with a UIP pattern on HRCT compared with other fibrotic patterns, but this trend did not reach statistical significance. Furthermore, the rate of decline in FVC was slower in RA-ILD patients treated with nintedanib rather than the placebo regardless of whether or not they were treated with background DMARDs or steroids at baseline. This suggests some synergistic treatment effect in combining DMARDs and nintedanib [46,47]. None of these trials show a clear statistically significant mortality benefit at 52 weeks. The long-term cumulative protective effect over multiple years, if indeed there is one, still needs to be demonstrated. Furthermore, health status questionnaires such as the King’s Brief Interstitial Lung Disease (K-BILD) questionnaire did not demonstrate a meaningful reduction in the composite of dyspnoea, chest symptoms and psychological well-being. There are also significant GI disturbance and diarrhoeal symptoms associated with the drug, as more than 60% of patients in these trials reported these side effects. It is still up for debate as to whether the 1-year marginal benefit of FVC preservation and the questionable long-term protective effect are enough to offset the lack of improvement in patients’ quality of life and the poor tolerability associated with nintedanib.

In terms of other anti-fibrotic agents, the TRAIL1 study was a phase 2 double-blind RCT that aimed to assess the efficacy and safety of pirfenidone in RA-ILD patients. Unfortunately, TRAIL1 was disrupted by the COVID-19 pandemic and ultimately only 123 patients were recruited, despite requiring 270 for an adequately powered study. The primary endpoint was the composite of a greater than 10% predicted decline in FVC % or death during the 52-week study period. This endpoint was met by 11% of the pirfenidone group versus 15% of the placebo group, suggesting a favourable protective effect against RA-ILD deterioration. However, given the difficulty with recruitment, the trial was underpowered to detect a statistically significant distance between groups in the composite endpoint [48,49]. Nevertheless, the reduction in the rate of decline in FVC at 52 weeks with pirfenidone vs. placebo in TRAIL1 is similar to that seen with nintedanib in INBUILD, at 55% and 60%, respectively. Furthermore, pirfenidone has been shown to reduce both IL-6 and TNF-α, which are central cytokines implicated in RA [50]. Lastly, pirfenidone has also shown an inhibitory effect on the transformation from fibroblast to myofibroblast in lung specimens from RA-ILD patients [51].

Ultimately, the body of evidence suggests that nintedanib likely has a synergistic effect when combined with DMARDs. The potential protective effect of pirfenidone in RA-ILD is a little less certain, although the trend is towards a beneficial effect. In practice, we would recommend the trial of an anti-fibrotic in those with progressive fibrotic lung disease, opting for nintedanib over pirfenidone, with commencement ideally after first monitoring the response to MTX and bDMARD (ABA, RTX, JAKi). If nintedanib is poorly tolerated from a gastrointestinal standpoint despite the use of loperamide, pirfenidone may be an alternative.

## 5. Complications in RA-ILD

Unsurprisingly, careful consideration must be given to the choice of immunosuppression on a risk–benefit case-by-case basis. Pre-existing lung disease and a poor pulmonary reserve prior to treatment, along with advanced age, make this a frail cohort of RA patients particularly susceptible to iatrogenic or immunosuppression-related infections. While much of the sequalae of long-term glucocorticoids can now be avoided with all the therapeutic classes available today, there remains an increased risk of bacterial pneumonias and class-specific risks. RTX may be the most suitable agent in those with a history of recent malignancy, but it has a particularly potent deleterious effect on vaccination response; in addition, given the long-lasting effect of a single infusion, one must be cautious of its use in frail patients prone to recurrent infections. In this sense, ABA may be a better option, but there is still a rare clinical scenario in which a prior history of melanoma would make it a poor choice. Lastly, PJP is probably the greatest concern in this cohort of frail patients. It can be difficult to diagnose given that the clinical picture can be confused with ILD exacerbation or a bacterial or viral LRTI superimposed on background pulmonary disease. Table 2 highlights the key differences and similarities between the two. Of particular concern is the high mortality rate associated with PJP infection. As a result, PJP is a main topic of discussion in terms of the potential complications encountered by RA-ILD patients.

### PJP and Prophylaxis

PJP is a serious and often underappreciated opportunistic infection in RA patients. While the incidence of PJP is lower in non-HIV patients, the associated mortality is considerably higher in RA-ILD patients on bDMARDs, at approximately 30% compared to 10–20% in HIV-positive patients [52,53,54]. Post-marketing surveillance studies report a PJP incidence rate in bDMARD-treated RA patients of 0.1–0.4% in Japan and 0.02–0.04% in Western countries [55,56,57,58,59].

Interestingly, the rate of the asymptomatic carriage of P. jirovecii in the RA population ranges from 10.9% to 28.5% [60,61,62]. As a result, there is little value to routine polymerase chain reaction (PCR) testing for PJP. PCR testing is not only expensive, but it may also be misleading given the high asymptomatic carriage rate, unless performed in the correct clinical context when PJP is suspected. The important risk factors that confer a high risk for PJP in the RA population are old age, high-dose steroids and pre-existing lung disease [63]. This makes the RA-ILD population relatively high risk. However, PJP prophylaxis carries a very real risk of myelosuppression, hepatotoxicity and Stevens–Johnson syndrome. Who, then, is a candidate for PJP prophylaxis?

First and foremost, the groups that benefit from PJP prophylaxis in general are those that are immunocompromised and are at a higher risk of PJP infection. Typically, PJP prophylaxis is recommended in HIV-infected patients with low CD4 cell counts, transplant recipients and those with haematologic malignancies. While the incidence rate of PJP is certainly lower in the RA-ILD population than in the aforementioned at-risk groups, the mortality rate in the RA-ILD cohort is alarmingly high. Katsuyama et al. showed that RA patients with two or three risk factors for PJP, such as steroid use, pre-existing lung disease and older age (>65), benefited from primary prophylaxis [64]. If initiating bDMARD treatment with high-dose prednisolone taper in an elderly RA-ILD patient, it is perfectly reasonable to prescribe low-dose PJP prophylaxis with the regular monitoring of the patient’s full blood count, liver function and renal profile. What is not practical, or indeed advisable, is an indefinite duration of PJP prophylaxis. Saito et al. showed that P. jirovecii disappears within 7–10 days after commencing trimethoprim–sulfamethoxazole (TMP–SMX) treatment in PJP patients with inflammatory rheumatic diseases without recurrence [65]. Additionally, Godeau et al. did not see any PJP relapses among survivors who had been treated for PJP infection with TMP–SMX for a mean of 17 days, despite recommencing their immunosuppressive regimens once recovered without secondary prophylaxis [66]. These studies tentatively support the idea that PJP prophylaxis may be discontinued after an initial period of treatment while on a tapering regimen of high-dose steroids. The use of low-dose PJP prophylaxis with TMP–SMX in RA-ILD patients should be considered if initiating a high-dose steroid taper in combination with a bDMARD.

## 6. RA-ILD Guidelines

There remains little by way of international consensus guidelines for the treatment of RA-ILD. The eagerly awaited American College of Rheumatology (ACR) guideline has yet to be formally published, but it has already raised eyebrows with its promotion of mycophenolic acid (MMF) as a first-line treatment and its heavy focus on nintedanib in the treatment algorithm. This guideline for the treatment of ILD in people with systemic autoimmune rheumatic disease (SARD) conditionally recommends MMF as the first-line therapy in RA-ILD, as well as the first-line treatment in all systemic-autoimmune-rheumatic-disease-associated ILD (SARD-ILD) [67]. The final form of this guideline is expected to be published in high-impact journals in 2024. While it is tempting to assume that the beneficial effect of MMF on ILD in dSSc, ANCA-associated vasculitis (AAV) and idiopathic inflammatory myopathies (IIMs) will carry over to RA-ILD, we are of the opinion that the evidence base to support the suitability of MMF as a first-line therapy in RA-ILD is weak.

**Figure 2:**
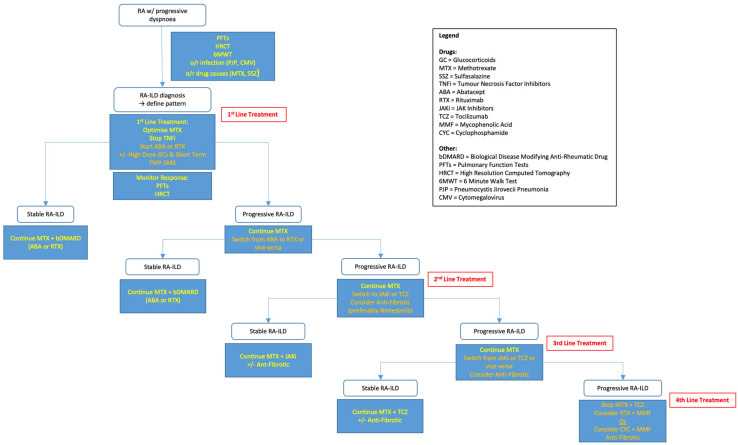
Proposed treatment algorithm for RA-ILD.

Currently, we believe that the Spanish Society of Rheumatology (Sociedad Espanola de Reumatologia or SER) has created and promoted the most suitable guidelines for RA-ILD based on the current available evidence base for each drug. The SER promotes individualised assessment for the use of MTX given the risk of drug-induced pneumonitis. They also advise the continuation of MTX in patients who have already been receiving the drug for more than one year at the time of RA-ILD diagnosis, as there is no evidence to justify discontinuation. ABA and RTX are the recommended bDMARDs of choice if required, and the SER does not promote one over the other. If there is an inadequate response to ABA or RTX, the SER recommends a switch to either TCZ or a JAKi; the SER does not prefer one over the other. If GCs are considered necessary in inflammatory non-UIP radiological patterns (NSIP, OP, LIP, etc.), then their use is recommended at the lowest dose and shortest duration possible [68]. Our proposed treatment algorithm in Figure 2 is very similar to the SER guidelines.

To the best of our knowledge, the BSR is currently in the process of developing its guidelines for the treatment of RA-ILD. It will be interesting to see whether the BSR’s new guidelines fall more in line with the SER or the ACR. We highlight and compare some of the key recommendations from the ACR, SER and BTS in Table 3.

## 7. Conclusions

While autoimmune parenchymal lung disease remains a significant cause of morbidity and mortality in RA patients, it is now clear that MTX has a protective rather than detrimental effect on ILD development. The current evidence base supports the avoidance or cessation of TNFi in those with newly diagnosed ILD, and the commencement of ABA or RTX preferentially. There remains a role for high-dose GCs in the acute setting and particularly in the presence of either the NSIP or OP pattern on HRCT. The short-term use of PJP prophylaxis is reasonable while tapering high-dose GCs. The emerging evidence suggests that the addition of an anti-fibrotic, preferentially nintedanib, has a synergistic protective effect. Ideally, combination MTX and bDMARD treatment should be optimised and monitored with follow-up PFTs (and HRCT, if access allows) before the addition of an anti-fibrotic. There remains a great need for further RCTs and prospective studies to help better determine the optimum treatment strategy in RA-ILD with the currently available drugs on the market.

## 8. Summary

1. MTX has a protective effect in RA-ILD patients and should be continued.
2. TNFi use should be avoided in RA-ILD patients.
3. ABA and RTX have the strongest evidence base and either should be added to MTX as a first-line bDMARD.
4. TCZ or JAKi may be considered as a second-line bDMARD in RA-ILD.
5. MMF may be considered in combination with RTX in refractory disease.
6. There remains a short-term role for high-dose GCs in NSIP or OP, but not UIP, patterns of RA-ILD.
7. Long-term GCs should be avoided.
8. PJP prophylaxis may be prescribed in the short term for a limited duration if on tapering high-dose GCs.
9. Nintedanib may be added after the optimisation of combination MTX + bDMARD (ABA or RTX).
10. Pirfenidone may be trialled if nintedanib + loperamide is not tolerated from a GI perspective.

## Figures and Tables

**Table 1:** Immunosuppressive therapeutics for RA-ILD.

Therapeutics in RA-ILD				
Drug	Indicated	Dose	Pre-Treatment Screening	Special Considerations
Glucocorticoids	Conditionally - Short Term - NSIP or OP	- 1 mg/kg - <3 months	n/a	- Caution in diabetics - Significant long-term sequelae - No role in UIP
Methotrexate	Yes	- 20–25 mg PO/SC weekly	- Hep B/Hep C	- Contraindicated in CKD IV, dose adjust in CKD III
TNF Inhibitors	No - Not optimal in new ILD or progressing ILD - Conditionally may continue if ILD stable on TNFi	n/a	- Hep B/Hep C - HIV1 and 2 - IGRA	- Uncertainty remains re effect on ILD - May be appropriate to continue
Rituximab	Yes - Best choice in recent malignancy	- 1 g IV every 6/12	- Hep B/Hep C - HIV1 and 2 - IGRA - IgG/IgM/IgA - SPEP	- Monitor IgG levels - Role of lymphocyte subset testing unclear - Timing of vaccinations important with rituximab
Abatacept	Yes	- 125 mg SC weekly - 500/750/1000 mg IV every 4/52 depending on weight	- Hep B/Hep C - HIV1 and 2 - IGRA	- Avoid if prior hx of melanoma
Tocilizumab	Yes	- 162 mg SC weekly - 8 mg/kg IV every 4/52	- Hep B/Hep C - HIV1 and 2- IGRA	- Monitor neutrophils and lipids - Not ideal in diabetics with leg ulcers
JAK Inhibitors	Yes	n/a	- Hep B/Hep C - HIV1 and 2 - IGRA - VZV	- Possible ↑ risk of major adverse cardiac event or thrombotic event
Mycophenolic Acid	Conditionally	1500 mg PO BD	- Hep B/Hep C - HIV1 and 2 - IGRA - VZV	- No effect on articular disease - Should be kept in reserve
Cyclophosphamide	Conditionally	Duration dependent	- Hep B/Hep C - HIV1 and 2 - IGRA - IgG/IgM/IgA - SPEP	- Last resort

**Table 2:** RA-ILD and PJP presentations and treatments.

Disease	RA-ILD	PJP in RA-ILD
Prevalence	Up to 10% of RA patients	0.1–0.4% of RA-ILD patients
Course	Insidious onset and course (can rarely be acute)	Acute or subacute onset
Signs and Symptoms	Non-productive cough Exertional Dyspnoea Fever Clubbing Bilateral Basal Crackles Rheumatoid Deformities Rheumatoid Nodules	Non-productive cough Exertional Dyspnoea Fever/Chills Chest Pain Fatigue (chest auscultation may be clear)
Investigations	RF ACPA Restrictive PFT Pattern	Sputum/BAL PCR ↑β-D Glucan ↑CRP ↑LDH
HRCT	Majority UIP NSIP OP	Diffuse GGOs
Poor Prognostic Factors	Older age Male gender ACPA+ High RF titre Subcutaneous nodules Smoker UIP pattern HLA-DR4B (DRB1*04) haplotype	Older age Male gender Steroids + bDMARDs (at time of PJP dx) Lymphopenia
Treatment	MTX + bDMARD (ABA or RTX) +/− Glucocorticoids acutely +/− Anti-fibrotics +/− PJP prophylaxis short term Consider TCZ, JAKi or MMF in refractory cases	TMP-SMX Alternatives: pentamidine, atovaquone, primaquine, clindamycin ± Glucocorticoids

**Table 3:** Key recommendations from RA-ILD guidelines.

RA-ILD Guidelines							
	Glucocorticoids	Methotrexate	TNFi	First-Line Agent	Second-Line Agent	Anti-Fibrotic	Other
ACR 2024	Conditionally, Yes - Short course - No comment on dose	No	No	MMF > AZA > RTX	AZA	No anti-fibrotic recommended as first line; may consider nintedanib if progressing	Ritux received fewer votes for first-line treatment than AZA
SER 2022	Conditionally, Yes - NSIP, OP, LIP - Lowest possible dose - Shortest possible duration	Yes - May continue	May continue if ILD stable	RTX or ABA	TCZ or JAKi	nintedanib recommended	CYC + MP advised in severe Non-UIP RA-ILD
BTS 2008	Yes - Prednisolone 0.5 mg/kg/day for 1–3 months - Maintenance dose of 10 mg/d or less	Conditionally, Yes - May consider continuing or adding to treatment	Warning against - No definitive advice	CYC - If failing GCs alone AZA D-PEN MTX	CIC	n/a	n/a

## Data Availability

Not applicable.

## Abbreviations

AAV	ANCA-Associated Vasculitis
ABA	Abatacept
ACPA	Anti-Citrullinated Protein Antibody
ACR	American College of Rheumatology
ADA	Adalimumab
AZA	Azathioprine
bDMARD	Biological-Disease-Modifying Anti-Rheumatic Drug
BSR	British Society of Rheumatology
BSRBB	British Society for Rheumatology Biologics Register
CIC	Ciclosporin
CMV	Cytomegalovirus
csDMARD	Conventional-Disease-Modifying Anti-Rheumatic Drug
CTD	Connective Tissue Disease
CTD-ILD	Connective-Tissue-Disease-Related Interstitial Lung Disease
CYC	Cyclophosphamide
DLCO	Diffusion Capacity of Carbon Monoxide
DMARD	Disease-Modifying Anti-Rheumatic Drug
D-PEN	D-Penicillamine
dSSc	Diffuse Systemic Sclerosis
EMA	European Medicines Agency
FDA	Food and Drug Administration
FVC	Forced Vital Capacity
GC	Glucocorticoids
HRCT	High-Resolution Computed Tomography
IIM	Idiopathic Inflammatory Myopathy
ILD	Interstitial Lung Disease
IL-6	Interleukin 6
JAKi	Janus Kinase Inhibitor
KL-6	Krebs Von Den Lungen-6 Level
LIP	Lymphoid Interstitial Pneumonia
MMF	Mycophenolic Acid
MTX	Methotrexate
NSIP	Non-Specific Interstitial Pneumonia
OP	Organising Pneumonia
PFTs	Pulmonary Function Tests
PJP	Pneumocystis Jirovecii Pneumonia
RA	Rheumatoid Arthritis
RA-ILD	Rheumatoid-Arthritis-Associated Interstitial Lung Disease
RTX	Rituximab
SARD	Systemic Autoimmune Rheumatic Disease
SARD-ILD	Systemic-Autoimmune-Rheumatic-Disease-Associated ILD
SER	Sociedad Espanola de Reumatologia
SSZ	Sulfasalazine
TCZ	Tocilizumab
TMP-SMX	Trimethoprim–Sulfamethoxazole
TNFi	Tumour Necrosis Factor Inhibitor
TOFA	Tofacitinib
UIP	Usual Interstitial Pneumonia
6MWT	6-Minute Walk Test
